# Supplementary material for: Van der Waals ferromagnetic Josephson junctions
Source: Nat Commun. 2021 Nov 12;12:6580. doi: 10.1038/s41467-021-26946-w (PMC8589954; doi:10.1038/s41467-021-26946-w)
Supplement: Supplementary file 1 — Supplementary Information [file 41467_2021_26946_MOESM1_ESM.pdf]

Supplementary Information for

Van der Waals Ferromagnetic Josephson Junctions

Linfeng Ai[1,2†], Enze Zhang[1†], Jinshan Yang[3†], Xiaoyi Xie[1,2], Yunkun Yang[1], Zehao Jia[1,2], Yuda Zhang[1,2], Shanshan Liu[1], Zihan Li[1], Pengliang Leng[1,2], Xiangyu Cao[1,2], Xingdan Sun[4], Tongyao Zhang[5], Xufeng Kou[6], Zheng Han[4,5], Faxian Xiu[1,2,7,8*], Shaoming Dong[3*]

[1] State Key Laboratory of Surface Physics and Department of Physics, Fudan University, Shanghai 200433, China
[2] Shanghai Qi Zhi Institute, 41th Floor, AI Tower, No. 701 Yunjin Road, Xuhui District, Shanghai, 200232, China
[3] State Key Laboratory of High Performance Ceramics & Superfine Microstructure, Shanghai Institute of Ceramics, Chinese Academy of Sciences, Shanghai, 200050, China
[4] Shenyang National Laboratory for Materials Science, Institute of Metal Research, Chinese Academy of Sciences, Shenyang 110016, China
[5] State Key Laboratory of Quantum Optics and Quantum Optics Devices, Institute of Opto-Electronics, Shanxi University, Taiyuan 030006, China
[6] School of Information Science and Technology, ShanghaiTech University, Shanghai 201210, China
[7] Institute for Nanoelectronic Devices and Quantum Computing, Fudan University, Shanghai 200433, China
[8] Shanghai Research Center for Quantum Sciences, Shanghai 201315, China

Contents：

1. Additional transport results of device #01
2. Discontinuity and irreversibility in I_c patterns
3. Excess Josephson current in thicker barrier device
4. Thickness-tuned crossover from Josephson coupling to quantum tunneling
5. Magnetic characterizations of $Cr_2Ge_2Te_6$
6. Barrier thickness dependence of Josephson junction parameters
7. Characterizations of single critical current due to the high damping environment in our Josephson junctions
8. Derivations of the magnetic field dependences of the flux and the interlayer's magnetization
9. Supplementary References

Supplementary Note 1. Additional transport results of device #01

Part 1. Josephson characterizations and Fiske steps

Additional transport properties of device #01 are shown here. Supplementary Fig. 1a is the temperature dependence of junction resistance (black) from 0.05 K to 300 K, and the calculated residual resistance ratio (RRR) at 10 K compared to 300 K is 0.15, which is of a similar magnitude to pure $NbSe_2$ (~ 0.12). A previous study in NbN/GdN/NbN JJ[1] showed that its junction behavior at high temperature is typical of a semiconducting barrier and then it turns into a continuous decrease in resistance below T_{Curie} of GdN, due to the exchange splitting of the tunnel barrier with reduction of effective barrier height for one spin sign. However, the decrease of resistance in our device is dominated by $NbSe_2$ themselves. We have summarized the data of all devices, for each performs a metallic behavior before its superconducting transition temperature. In line with the small hysteresis of the *IV* curves as well as small R_n, we have to address that the $Cr_2Ge_2Te_6$ has a small gap that can be doped easily by the environmental charge impurities. There's a high possibility that at the base temperature there is still some residual spin-polarized density of states (DOS) in the tunneling barrier itself.

Supplementary Fig. 1b presents the *IV* curves measured at the different magnetic fields as labeled in the inset, and the arrows indicate two current steps at bias voltage because of the Fiske resonance. In device #01, we can see a first-order Fiske step in which the positions of the 2nd maximum in I_c correspond to the 1st minimum. Besides, a second-order Fiske step occasionally appears in device #02, as shown in the main text Fig. 4a. The voltage position of the first Fiske step V_1 is 0.57 mV both indicated in the 4 mT (yellow) and 9.5 mT (blue) curves. The two-gap edge state of JJ device #01 is obtained from the integral of the *dV*/*dI* curve, and the value V_2 of ~ 2 mV is the same as the SIS tunnel junction results in the following sections.

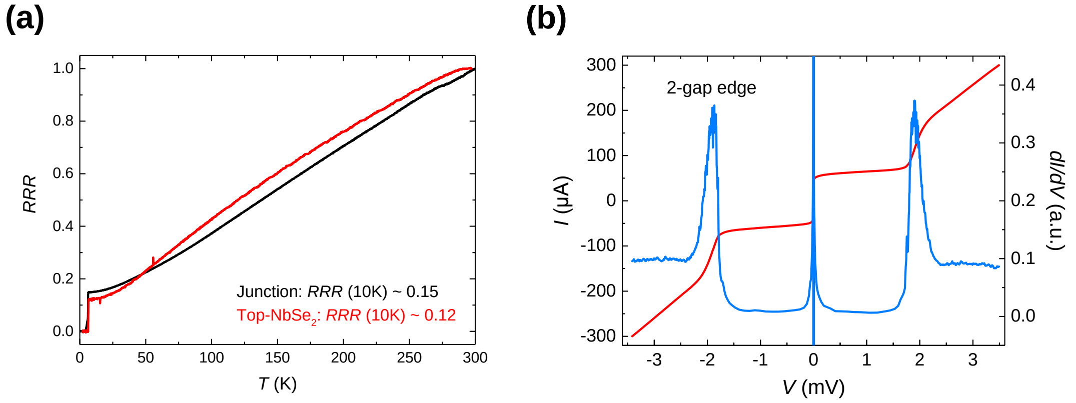

Supplementary Fig. 1. Additional JJ characteristics of device #01. (a) Temperature dependence of resistance of the junction (black) and $NbSe_2$ (red) from 0.05 K to 300 K, which shows a metallic barrier behavior above T_c. **(b)** Differential conductance (blue) along with charge current (red) as a function of the bias voltage. The two-gap edge state value is ~ 2 mV, twice the gap of the superconductor electrodes ($NbSe_2$).

(a)

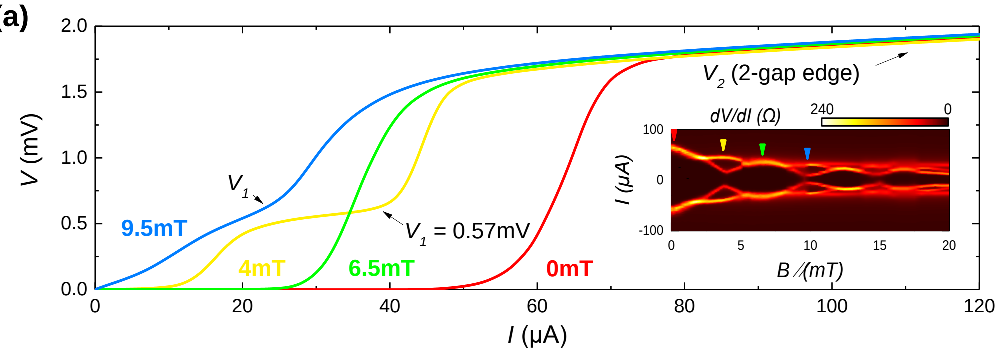

Supplementary Fig. 2. Fiske steps characteristics of device #01. (a) *IV* curves measured at the different magnetic fields to reveal the Fiske steps. The inset shows a Fraunhofer pattern of device #01 with first-order Fiske steps (V_1 = 0.57 mV) labeled by the colored arrows.

Indeed the phenomenon of Fiske resonance is more essential driven by the voltage rather than the current. According to the Josephson equations, the supercurrent across a JJ oscillates at a constant frequency $\omega_J = 2\pi V/\Phi_0$ at an applied voltage V[2]. When the oscillation frequency matches the *n*th harmonic of the JJ cavity mode as the spatial period of the Josephson current distribution matches the spatial period of the *n*th resonant electromagnetic mode of the junction, achieved by applying a magnetic field, it will result in the excitation of the cavity modes as the self-induced current steps at specific voltages V_n denoted as Fiske steps[3–5]. Regrettably, we have used the current bias to drive the JJ in our experiments and mainly focused on its critical current, therefore we did not try the voltage-driven operations to reproduce the Fiske resonance for a comparison. Nevertheless, we try to integrate the current into the voltage coordinate by the differential resistance *dV*/*dI* to reveal the complementary details of the observed Fiske steps results.

The comparison between the Fiske steps' configuration driven by the current and the voltage is presented in Supplementary Fig. 3a. We can see that in the lower panel, the shaded areas indicated by the black and white dashed circles represent the specific excitation voltages V_1 and V_2 corresponding to the first and second-order Fiske resonances respectively. Although the original current bias acting as the coordinate has relatively higher accuracy in determining the peak positions of *dV*/*dI*, we can only roughly define the resonant modes in its integration plots because of the highly damping environment in our JJs as Supplementary Fig. 3b showed, where the resonances are not sharp enough at the almost constant voltages. The value of V_1 is defined as the valley position of *dV*/*dI* vs *V* at 0.57 mV (Supplementary Fig. 4b), and the consequent specific capacitance C_s and the parameter β_c mentioned in the main text can be calculated for estimations.

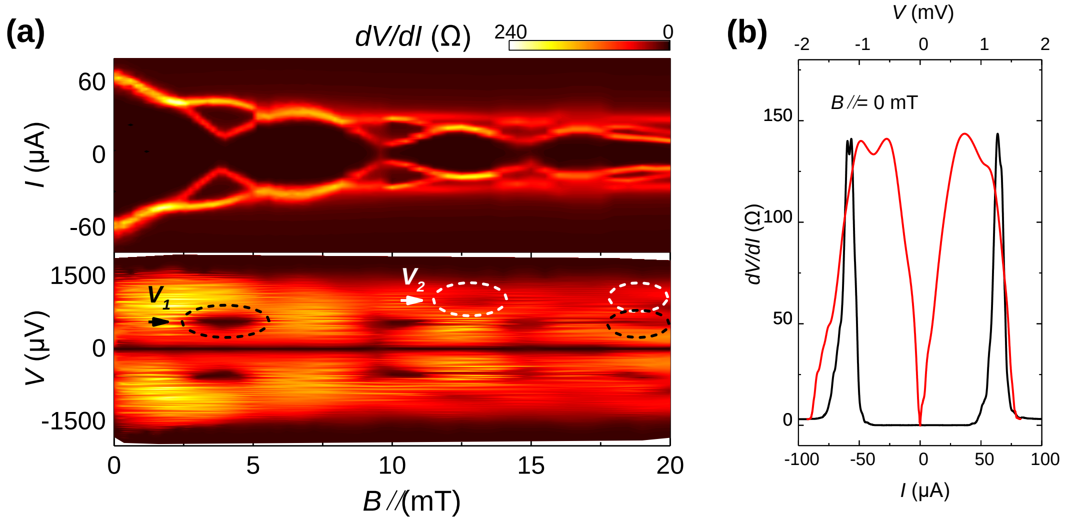

Supplementary Fig. 3. Fiske resonances in device #01 driven by the voltage. (a) Comparison of signatures of the Fiske resonances in the current (upper panel) and the voltage (lower panel) coordinate. The first and the second-order Fiske steps are labeled by the black (V_1) and white (V_2) arrows with slight broadening due to the errors of integration. **(b)** dV/dI as a function of current (black) and its integrating voltage (red) measured at zero magnetic field.

Next, more characterizations are presented to confirm the flux dependence of the resonant modes, in which we select five line cuts of the voltage-driven plots at the different magnetic fields in the case of successive periods of its diffraction pattern, indicated as Supplementary Fig. 4a. The valley positions of the curves measured at 4, 9.5 and 15 mT shown in Supplementary Fig. 4c are nearly the same regardless of slight shifts, accounting for the clear first-order Fiske resonance at around 0.57 mV. The second-order Fiske resonance is observed in the curve of 12.5 mT at around 0.97 mV while the first-order step disappears. Compared to the single resonant mode results, they simultaneously occur in the curve of 18.5 mT shown in Supplementary Fig. 4d, and the resonance voltages are very close to the values above. Therefore we can find it easier to identify the Fiske resonances in the voltage-driven plots as the reviewer suggested. To precisely determine the resonant voltages, we expect further improvements in the devices' qualities for negligible damping circumstance and higher-order Fiske steps, under which the internal losses in our JJs can be revealed more accurately.

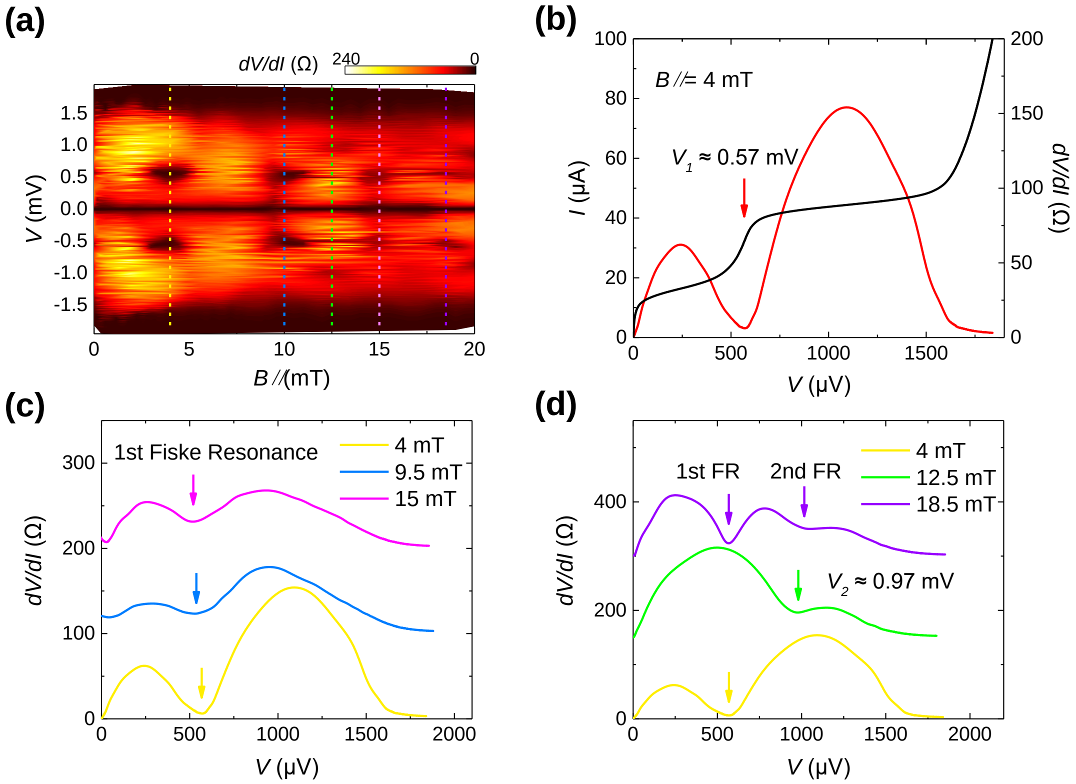

Supplementary Fig. 4. Flux dependence of the resonant modes driven by the voltage in device #01. (a) Fiske resonances in the voltage coordinate. The dashed lines mark out the positions of successive quantum fluxes embedded in the junction (yellow: 4 mT for Φ_0; blue: 9.5 mT for $2\Phi_0$; green: 12.5 mT for $2.5\Phi_0$; pink: 15 mT for $3\Phi_0$; purple: 18.5 mT for $4\Phi_0$). **(b)** I (black) along with dV/dI (red) as a function of bias V measured at 4 mT in-plane magnetic field. The dip in its dV/dI represents the first order Fiske step at V_1 = 0.57 mV. **(c)** dV/dI vs V curves measured at 4, 9.5, and 15 mT, where the orders of Φ_0 equal to integers and only the first Fiske resonant mode can be seen. **(d)** dV/dI vs V curves measured at 4, 12.5, and 18.5 mT, where the second Fiske resonant mode V_2 is at about 0.97 mV.

Part 2. Magnetic response at low temperature

A detailed response to the parallel magnetic field in device #01 is shown in the next parts. Supplementary Fig. 5a shows the magnetoresistance results measured at 1.9 K. For the junction, its resistance increases below 60 mT and eventually decreases up to 9 T when spins in the intermediate $Cr_2Ge_2Te_6$ were aligned to the field direction. In the meanwhile, the Ising superconductors $NbSe_2$ with a large parallel critical field (at the top and bottom) were still in the superconducting state with zero resistance, suggesting that the entire contribution of junction resistance comes from the tunneling barrier. In Supplementary Fig. 5b we show that the illustrations of barrier moment as a sequence of applied field split into three stages from the junction resistance, which corresponds to a complete hysteresis magnetization loop of magnetic barriers.

During the experiments, we have used two measurement systems to carry out the transport data in different temperature ranges, therefore a comparison between them is

needed. From the arrows in Supplementary Fig. 5c we can see the central positions of two sweep branches are slightly shifted, meanwhile, the oscillations at 0.1 K are evident, compared to 1.9 K due to fewer thermal perturbations. Nevertheless, the magnitude of hysteresis is almost the same at these two temperatures (for one starts from 30 mT and the other from 27 mT), which also proves the validity that we use $\Delta B_{\max}$ as an index for estimation from 7 K to 0.1 K.

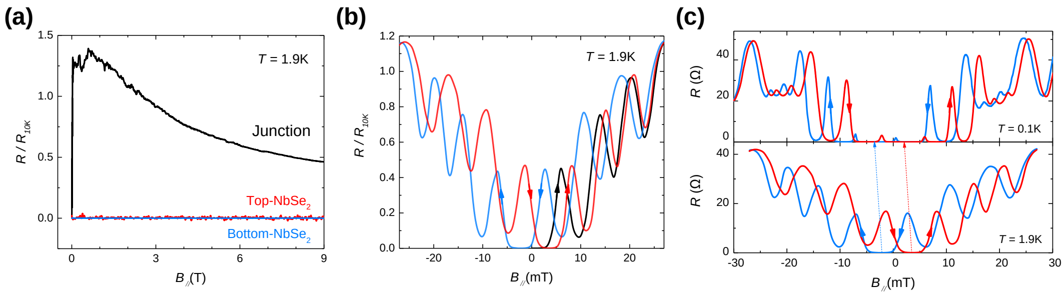

Supplementary Fig. 5. Additional magnetic response of device #01. (a) Magnetoresistance of the junction (black) and pure superconductor layers (red for the top and blue for the bottom) versus parallel magnetic field up to 9 T. **(b)** Oscillatory resistance patterns of the junction at 1.9 K. The black line is measured after zero-field cooling to induce an initial magnetization of the barrier, and the blue and red curves are responses in downward and upward directions of field sweeping, respectively. **(c)** Comparison of hysteretic resistance patterns between the two measurement systems in different temperature ranges (0.1 K in a dilution refrigerator and 1.9 K in a PPMS).

Since the hysteresis introduced by the magnetic barrier in JJs can be controlled by $B_{\max}$, we show a stacked plot of hysteretic junction resistance with $B_{\max}$ ranging from 20 mT to 100 mT in Supplementary Fig. 6a. The coincidence of the opposite direction sweeping curves over 75 mT shown in the bottom indicates a saturated magnetized state, in contrast to smaller $B_{\max}$ situations that the shift between the two curves arises immediately as the field direction turns back. Consequently, with a step-increase of $B_{\max}$, the central lobe (equivalent to a zero-flux state in the junction) moves as guided by arrows in Supplementary Fig. 6a, and the variation of its peak positions at downward or upward sweeping is presented in Supplementary Fig. 6b (1L represents the 1st maximum resistance at the left side of the central lobe while 1R at right side). Supplementary Fig. 6c depicts the separations between the two sweeping branches at different $B_{\max}$, from which we can clearly see a rapid increase in measured hysteresis since the initial magnetization, and then it gradually converges above 75 mT, the same as the conclusion reached from Supplementary Fig. 6a.

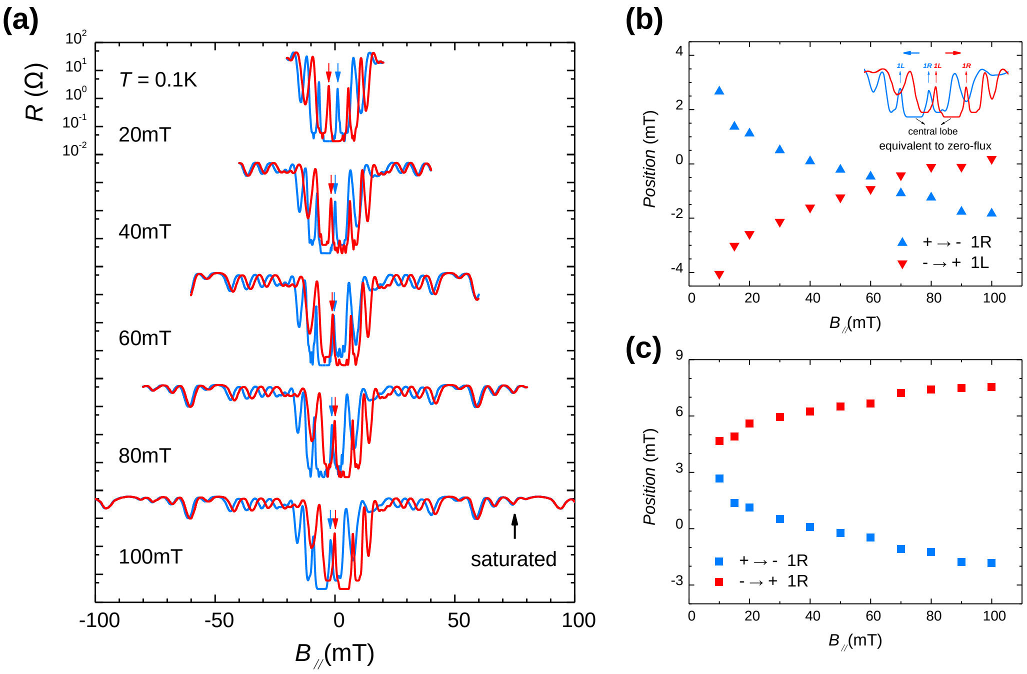

Supplementary Fig. 6. Junction magnetoresistance at different in-plane B_{max} of device #01 at 0.1K. (a) Log-plot of junction magnetoresistance measured with B_{max} ranging from 20 mT to 100 mT at 0.1 K (blue curves for downward while red ones for upward). The barrier moment becomes saturated over 75 mT. **(b)** The variation of the two sweeping branches is shown in Supplementary Fig. 6a. Inset: illustrations of the central lobe (equivalent to a zero-flux state in the junction) and peak 1L/1R in downward (blue) and upward direction (red), respectively. **(c)** The barrier's hysteresis evaluated from the corresponding peak 1R positions both for downward and upward directions.

Part 3. Junction resistance near T_c and high *d.c.* bias

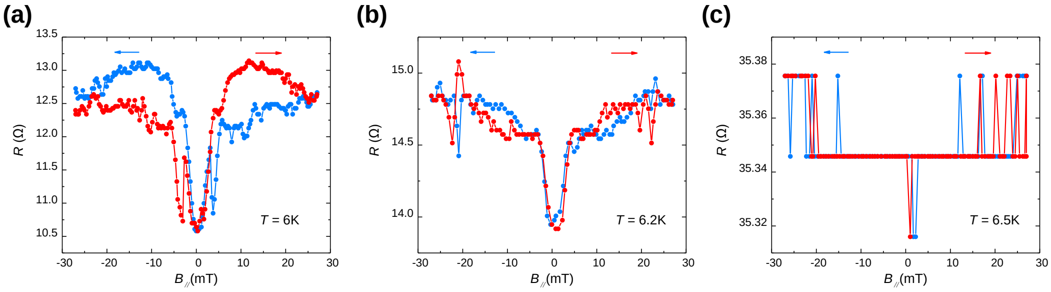

Supplementary Fig. 7. Junction magnetoresistance of device #01 near T_c. (a-c) Junction resistance (blue for downward and red for upward) measured at 6 K, 6.2 K, and 6.5 K. The discrepancies in the center positions of the curves tend to be illegible as the temperature approaches T_c, which causes a sudden drop of ΔB_{max} value as the data shown in the main text Fig. 3(d). When the temperature is above T_c (~ 6.4K), measured hysteresis is almost zero within the error of field resolution.

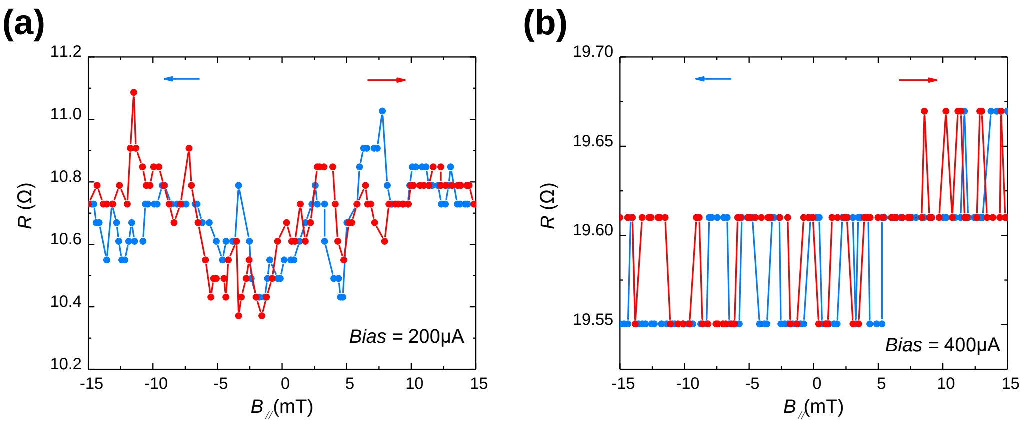

Supplementary Fig. 8. Junction magnetoresistance of device #01 at high *d.c.* bias. (a-b) Junction resistance measured at the *d.c.* bias of 200 μA and 400 μA. The deviations here are also practically unidentifiable at a high bias when the superconductor mainly contributes to the junction resistance.

Supplementary Note 2. Discontinuity and irreversibility in I_c patterns

Part 1. Discontinuity in device #01

The breakpoints that appeared in I_c patterns arise with the progressive magnetization of a highly polarized barrier, manifested as the abrupt jumps of I_c evidently at higher field. In Supplementary Fig. 9, we first show the breakpoints measured in device #01 and the *dV*/*dI* curves on both sides of the breakpoint near 25 mT. The conspicuous discontinuities above 23 mT are in sharp contrast to the patterns at the lower field region (the single step of the applied field is 0.5 mT in the whole process). Nevertheless, the results in the higher field become less recognizable because of the non-zero junction residual resistance at zero bias, thus we cannot get a valid I_c value there.

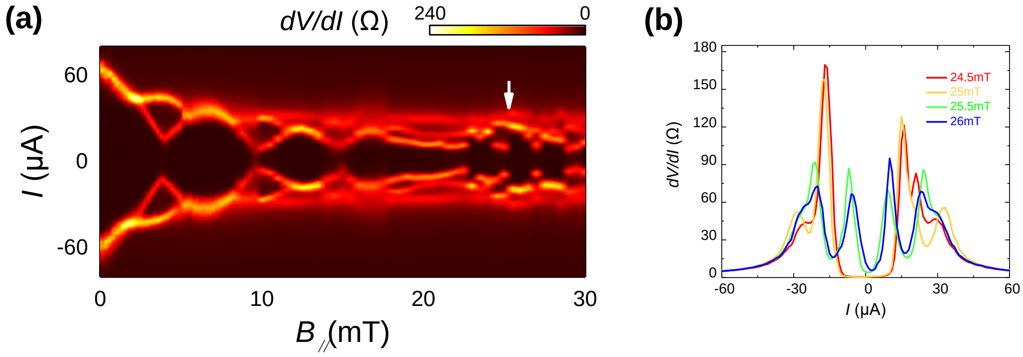

Supplementary Fig. 9. Illustrations of discontinuity in device #01. (a) The Fraunhofer pattern of device #01 initially magnetized to 30 mT at 0.1 K, where the

discontinuity in I_c starts from 23 mT. **(b)** *dV/dI* curves under different magnetic fields near the breakpoint at 25 mT (indicated by the white arrow in Supplementary Fig. 9a). The I_c measured at 24.5 and 25 mT are almost identical (10.5 μA), but then encounter a sudden drop to almost zero as measured at 25.5 and 26 mT. The step of field resolution is 0.5 mT.

Part 2. Discontinuity and irreversibility in device #02

In device #02, the zero resistance state persists till -140 mT, in case that we can observe its first appearance of breakpoints at around -90 mT (Supplementary Fig. 10a). When the field reverses back to zero, the breakpoints still maintain their dominance even in the range where I_c has previously varied continuously (Supplementary Fig. 10b). Apart from the interlayer coupling in the $Cr_2Ge_2Te_6$ barrier which prevents the flip back of spins as we have discussed in the main text, the assumptions on multi-domains state possessing discrepant coercivity in the junction region may also be suggested to explain the step-like behaviors in I_c.

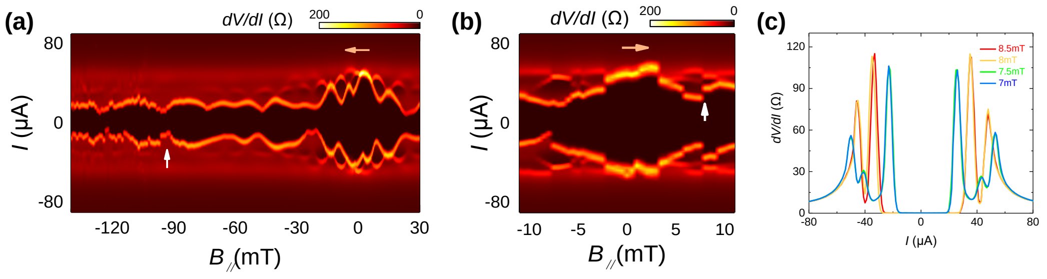

Supplementary Fig. 10. Illustrations of discontinuity and irreversibility in device #02. (a) Fraunhofer-like oscillation patterns of device #02 ranging from 30 mT to -140 mT at 0.1 K, where the breakpoints in I_c firstly appear at around -90 mT (marked by the white arrow). **(b)** Remeasured I_c patterns near zero magnetic field after reversing back from -140 mT, presents pronounced irreversibility. **(c)** *dV/dI* curves under different magnetic fields near the breakpoint at 8 mT, which is pointed by the white arrow in (b).

Supplementary Note 3. Excess Josephson current in thicker barrier device

For a thinner barrier like device #01 (~ 6.5 nm), the tunneling behavior shows a good coincidence to the AB relation, whose general form implies an invariance of I_c and the R_n depends on the measured superconducting energy gap. Note that the R_n is ~ 10.6 Ω obtained from the linear part in its *IV* curves and the fitting results are shown in Supplementary Fig. 11a. The calculated I_cR_n product (V_c) at zero temperature is ~ 0.51 mV, which the magnitude is much smaller than the theoretical value of $\pi\Delta_0/2e$ = 1.57 mV (bulk $NbSe_2$ energy gap: Δ_0 ~ 1 meV). The strong suppression shows that the tunneling probability of pairs in a magnetic barrier is lower than that for single electrons.

However, in a thicker barrier device #03 (~ 11nm), we find an apparent excess Josephson current substantially deviated from the AB relation at low temperature. The

data and fitting results (with a data range of 4.6 K to 6 K) are shown in Supplementary Fig. 11b. The I_cR_n product obtained at the base temperature of 0.5 K is 1.43 mV, exceeding the diffusive limit from the AB relation fit at zero temperature (0.84 mV). We tend to attribute it to a possible multi-domain structure in few-layer $Cr_2Ge_2Te_6$ especially for thicker layers, and the introduced magnetic inhomogeneity by the relative size of domain walls implies a reduction of exchange energy in the ferromagnets, which accounts for the enhancement.

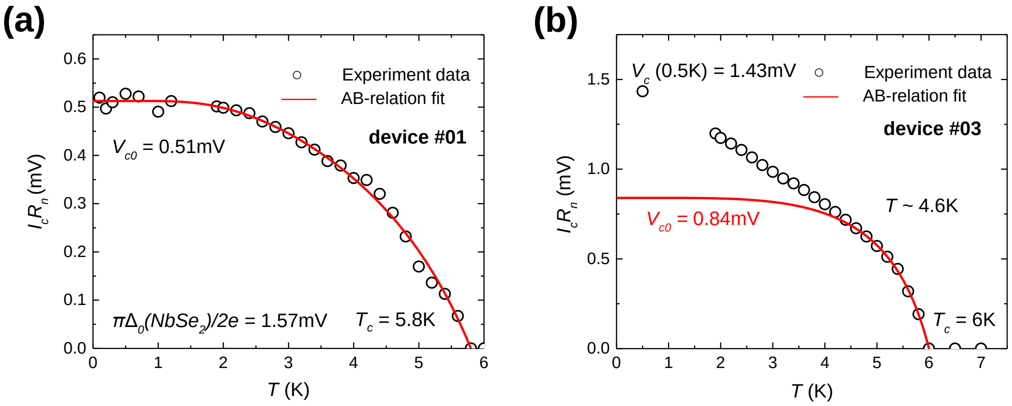

Supplementary Fig. 11. Multi-domains induced excess Josephson current in the thick barrier device. (a) Temperature dependence of I_cR_n (V_c) product of device #01 (black open circles) with a 6.5 nm barrier, and the red solid line is the fit to AB relation for an insulating barrier which gives V_{c0} = 0.51 mV. The theoretical value is ~ 1.57 mV for $NbSe_2$ JJ. **(b)** Temperature dependence of I_cR_n product of device #03 with an 11 nm barrier and the largest V_c acquired at 0.5 K is 1.43 mV. The red solid line is an AB relation fit from 4.6 K to 6 K which gives V_{c0} = 0.84 mV. The deviation between measured data and the fit is originated from the multi-domain structure in few-layer $Cr_2Ge_2Te_6$ especially for thicker samples, where the magnetic inhomogeneity can induce an equivalent reduction of exchange energy E_{ex}.

Supplementary Note 4. Thickness-tuned crossover from Josephson coupling to quantum tunneling

In addition to the JJ devices with relatively thin $Cr_2Ge_2Te_6$ as barriers, we have also investigated the crossover from the Josephson coupling state to the quantum tunneling dominant situation tuned by the magnetic barrier thickness. For a thick insulating layer, the Cooper pair wavefunction Ψ penetrating the JJ has damped to zero in the middle of the barrier that cannot provide a stable phase coherence, thus the transport conductance of the junction here is dominant by the quantum tunneling effect through single-electron channels[6] (Supplementary Fig. 12a).

In the left inset of Supplementary Fig. 12b, we first display the normalized differential tunneling conductance (G_s/G_n) at 2 K for a 14.5±0.4 nm thick $Cr_2Ge_2Te_6$ junction device #05. The separation between the peaks corresponds to the superconducting gap of bi-lateral few-layer $NbSe_2$ (20 ~ 40 nm), whose properties are similar to the bulk $NbSe_2$. For the quantitative analysis of the tunneling spectra, we

use the Blonder-Tinkham-Klapwijk (BTK) model[6] for SIS tunnel junctions with arbitrary barrier strength. Here, the temperature dependence of the gap (Supplementary Fig. 12b) is described by the BCS theory, and the extracted zero-temperature gap value is $2\Delta_0$ = 1.98 meV quantitatively consistent with the value 3.53 k_BT_c (k_B is the Boltzmann constant and T_c ~ 6.7 K is consistent with the resistance measurements). Dimensionless effective barrier strength Z is 0.87 at 2 K, which is comparable to other vdW tunneling materials[7,8].

The magnetic response of the tunnel junction versus the in-plane field is shown in Supplementary Fig. 12c, at zero bias voltage. The resistance is normalized to the value at different temperatures in the absence of field. The hysteresis shrinks in resistance amplitude and magnetic field range with increasing temperature, albeit it fully disappears above T_c, similar to what we have observed in JJ devices. It's also noteworthy that the junction resistance near zero field is smaller than that in the higher field, and such a dip possibly accounts for a lower effective barrier height corresponding to zero magnetization compared to the fully-polarized state, which may also be influenced by the two spin-mixed S/F heterointerfaces to generate an anisotropic magnetoresistance[9,10].

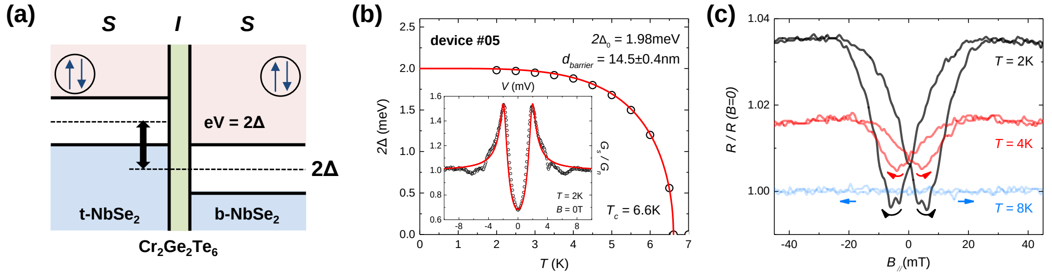

Supplementary Fig. 12. Quantum tunneling results of thicker barrier devices. (a) Schematic views of the energy diagram of the SIS tunnel junction with the same superconductors, showing that the conductance as a function of bias voltage measures the BCS superconducting gap 2Δ. **(b)** Normalized differential tunneling conductance (G_s/G_n) at 2K under zero field for a thick barrier device #05 (~ 14.5nm), and the BTK fitting gives the zero temperature BCS gap $2\Delta_0$ for $NbSe_2$ of 1.98 meV (black opened circles for experiment data and red solid lines for fit curves). **(c)** In-plane magnetoresistance of device #05 at 2 K, 4 K, and 8 K compared among the superconducting state and normal state.

Our results provide a barrier thickness-tuned crossover from quantum tunneling to Josephson coupling in $NbSe_2/Cr_2Ge_2Te_6$ devices, nevertheless, we cannot confirm that it is a continuous process. Both of the effective penetration length and surface impurities are required to accomplish an entire zero resistance state in a highly transparent and defect-free junction, therefore we plot a summary of normalized residual resistance (R_{2K}/R_{10K}) varied with barrier thickness in the range of 6 - 15 nm. The results are divided into three regions: $R_{2K}/R_{10K} > 1$, $R_{2K}/R_{10K} = 0$ and intermediate step $0 < R_{2K}/R_{10K} < 1$. All the devices' resistance in the Josephson coupling regime (6 ~ 11 nm) continuously decreases when cooling down, in sharp contrast to the

tunneling devices (13 ~ 15 nm), however, for the others (11 ~ 13 nm) the junction resistance saturates to a finite non-zero value at low temperatures. A careful comparison of their temperature dependences is shown in Supplementary Fig. 13a. We suppose that the junction resistance is formed by a series connection of two $NbSe_2$ electrodes and the non-superconducting $Cr_2Ge_2Te_6$ part, while the former is zero below T_c and the latter is the main cause of residual resistance. It is suggested that the effective penetration length of Ψ in the dirty limit should be comparable to the critical thickness for Cooper pair tunneling in these magnetic JJs. Interfacial scattering and impurities will lead to a finite residual resistance value even in JJ samples of thinner barriers, as suppressions of penetration length which is equivalent to thicker barriers.

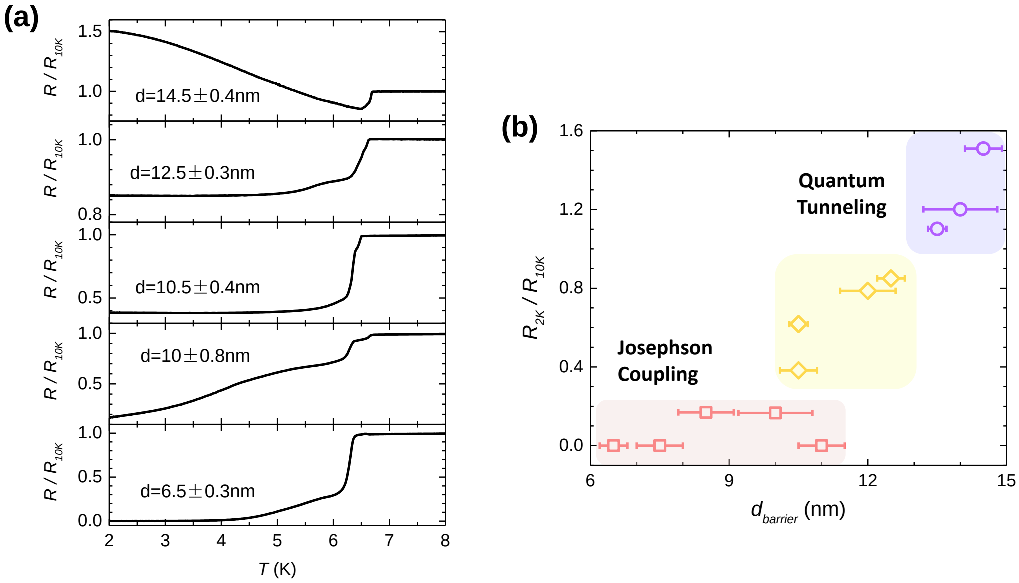

Supplementary Fig. 13. Summary of thickness-dependent tunneling process. (a) Temperature dependence of different devices with a thickness of 6.5, 10, 10.5, 12.5, and 14.5 nm, respectively. The residual resistance measured at 2 K increases from 0 to over 1 suggesting the crossover from the Josephson coupling state to the quantum tunneling dominant state. **(b)** Normalized residual resistance at 2 K when tuning the $Cr_2Ge_2Te_6$ barrier thickness, in considerations of interface impurities and defects.

Supplementary Note 5. Magnetic characterizations of $Cr_2Ge_2Te_6$

The birth of microscopic magnetic domain structure in $Cr_2Ge_2Te_6$ is related to the competition between the magnetocrystalline anisotropy and magnetic dipolar interaction, where the latter one always prefers an in-plane magnetization and becomes significant in low-dimensional materials[11]. A multi-domain state is favored in thick ferromagnetic films due to the dipolar energy winning over the exchange energy[12], while in some cases for ultrathin flakes whose easy axis is perpendicular to the film, dipolar interaction is expected to be very small, probably leading to a very

large domain size as shown in a six-layer $Cr_2Ge_2Te_6$ from Gong et al's results[13]. The dependence of the domain size (D) should both take the variety of domain wall energy (U_w) and also the film thickness (L) comparable with the range of exchange interactions (r_e) into account. The model put forward by Kooy and Enz[14] predicted that the domain size is proportional to the film thickness when L is much larger than the characteristic 'dipolar length' (D_0), however as L is reduced below D_0 while still larger than r_e, Kaplan and Gehring[15] found that the domain size will dramatically increase as film thickness decreases in the ultrathin regime. Taking the magnetic parameters of $Cr_2Ge_2Te_6$ as shown above, its domain wall energy density (σ_w) and the domain wall size can be estimated[16]

$$\sigma_w = 4\sqrt{J_x(K_u - \frac{1}{2}\mu_0 M_s^2)},\ D = \pi\sqrt{J_x/(K_u - \frac{1}{2}\mu_0 M_s^2)}.$$

The characteristic parameters of $Cr_2Ge_2Te_6$ are obtained from several previous papers (J ~ 3.76 meV [ref. [13]] and the consequent exchange stiffness $J_x \sim 1.53\times10^{-11}$ J m^{-1}, K_u ~ 3.3-3.7$\times10^4$ J m^{-3} [refs [17–19]] and M_s ~ 2-3 μ_B/Cr [refs [20–22]]). Therefore, the domain size of the $Cr_2Ge_2Te_6$ is calculated to be in the range of about 60-90 nm, and the consequent dipolar length $D_0 = 2\sigma_w/\mu_0 M_s^2$ is around 3-13 μm, far greater than the thickness of ferromagnetic film L. Meanwhile, the range of exchange interactions in $Cr_2Ge_2Te_6$ is evaluated by the distance of neighboring Cr atoms, so the conditions required in the model above could be satisfied. Except for the theoretical estimations, we still want to discuss the single-domain or multi-domain structures in $Cr_2Ge_2Te_6$ nanoflakes or bulks observed in previous experiments.

In Gong et al's results[13], the single-domain remanence is evident in ultrathin $Cr_2Ge_2Te_6$ ranging from 2-6L through Kerr rotation measurements, in contrast to the formation of multi-domains usually in the bulk. For comparison, the signatures of a multi-domain state have also been reported as the slanted Kerr rotation hysteresis loops of a 19 nm $Cr_2Ge_2Te_6$ sample from Wang et al's results[23], as well as the slanted induced Anomalous Hall Effect (AHE) signals in a 35 nm $Cr_2Ge_2Te_6$/Pt device from Lohmann et al's results[12]. For much thicker $Cr_2Ge_2Te_6$ samples (91-301 nm), Han et al[24] have observed stripe-like and bubble-like multiple domain structures assisted by the Lorentz transmission electron microscopy (LSTM). Also in the bulk $Cr_2Ge_2Te_6$, Guo et al[25] have found various multiple domain structures and symmetries stem from the competition between thermal fluctuation and anisotropy energy induced by magnetic dipolar interaction through magnetic force microscope (MFM) measurements. From the known observations of multi-domains in $Cr_2Ge_2Te_6$ nanoflakes or bulks, the characteristic domain size is around 70~120 nm, which is much larger than the few-layer $Cr_2Ge_2Te_6$ nanoflake thickness (<10 nm). To verify the magnetic properties of our $Cr_2Ge_2Te_6$ flakes with those reported, we have further performed some characterizations to give a comprehensive understanding.

We first characterized the intrinsic magnetic properties of $Cr_2Ge_2Te_6$ bulk crystals, where the magnetization measurements are performed through a SQUID magnetometer. The Curie temperature T_c ~ 63 K was determined from the drop in the temperature dependence of its magnetization when a 100 mT magnetic field is applied to the in-plane (along a-b plane) and the out-of-plane (along c axis) of the crystal, as

shown in Supplementary Fig. 14a. The bulk magnetizations below T_c with an external magnetic field reveal a ferromagnetic behavior with magnetic anisotropy by the comparison of their saturation fields measured at 2 K, as shown in Supplementary Fig. 14b. With a larger saturation field along the in-plane direction, the $Cr_2Ge_2Te_6$ crystal shows an easy axis perpendicular to the a-b plane, in agreement with previous reports[17,26].

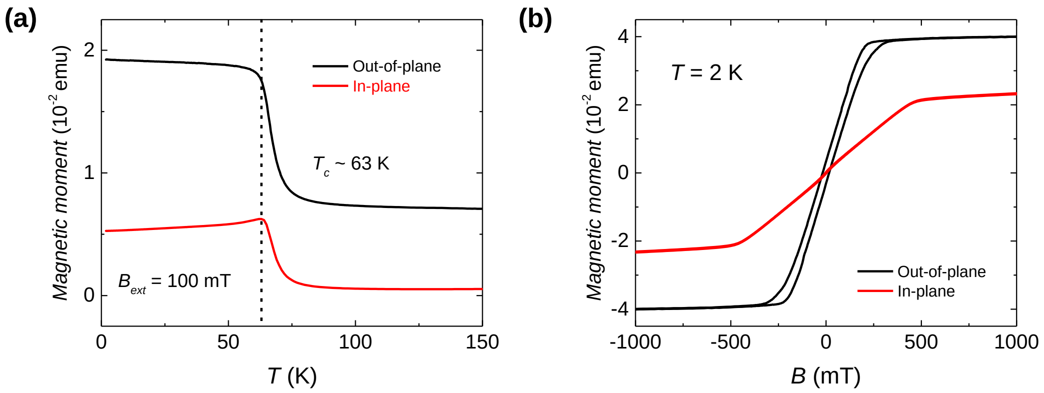

Supplementary Fig. 14. Magnetic characterizations of bulk $Cr_2Ge_2Te_6$ by SQUID magnetometer. (a) Temperature dependence of the bulk $Cr_2Ge_2Te_6$ sample's magnetization with a 100 mT magnetic field applied along the out-of-plane (black) and in-plane (red) directions. **(b)** Field dependence of its magnetizations at 2 K (black curves for out-of-plane direction and red curves for in-plane direction).

To further confirm the magnetic behavior in exfoliated $Cr_2Ge_2Te_6$ nanoflakes, we performed the Kerr rotation measurements to explore their properties changing with layer thickness. The measured temperature is at 5 K, below the critical temperature of $NbSe_2$ which is used as a superconducting electrode in our van der Waals (vdW) Josephson junction (JJ) devices. The original Kerr rotation signals under the out-of-plane magnetic field are present in Supplementary Fig. 15a, where we can obtain that the saturation magnetizations of $Cr_2Ge_2Te_6$ nanoflakes increase as the film thicknesses increase. In Supplementary Fig. 15b we present the normalized curves extracted from Supplementary Fig. 15a for clarity in comparison, and we can clearly see that for thinner $Cr_2Ge_2Te_6$ samples like 4 nm and 12 nm flakes, they only show monotonic transitions in the Kerr rotation hysteresis loops. Besides, the magnetic anisotropy is more obvious in the thinnest 4 nm $Cr_2Ge_2Te_6$. Yet a slanted behavior, as well as multiple transitions from the Kerr rotation loops under lower magnetic field, appears in the 20 nm $Cr_2Ge_2Te_6$ sample, suggesting the formation of nonuniform magnetizations in this thickest $Cr_2Ge_2Te_6$ flake, probably arisen from such as multi-domains. Combined the results reported before with our characterizations on $Cr_2Ge_2Te_6$ samples, both the larger domain size up to 10 μm as a single-domain remanence in ultrathin nanoflakes and the smaller domain size around 100 nm along with a multi-domain structure in much thicker $Cr_2Ge_2Te_6$ samples or even bulk crystals have been confirmed. Although we haven't found the counterexamples in these two thickness regions for $Cr_2Ge_2Te_6$, we cannot conclude a solid correlation

between the equilibrium domain size of $Cr_2Ge_2Te_6$ with its thickness, nor could we prove the tendency of forming multi-domains as the film thickness increases approaching a 3D ferromagnet up to our considerations. The basic purpose to give such an argument is trying to explain the enhancement of Josephson current in the thick-barrier device, therefore we will only limit our discussions to the range of $Cr_2Ge_2Te_6$ thickness below 20 nm according to our experiments.

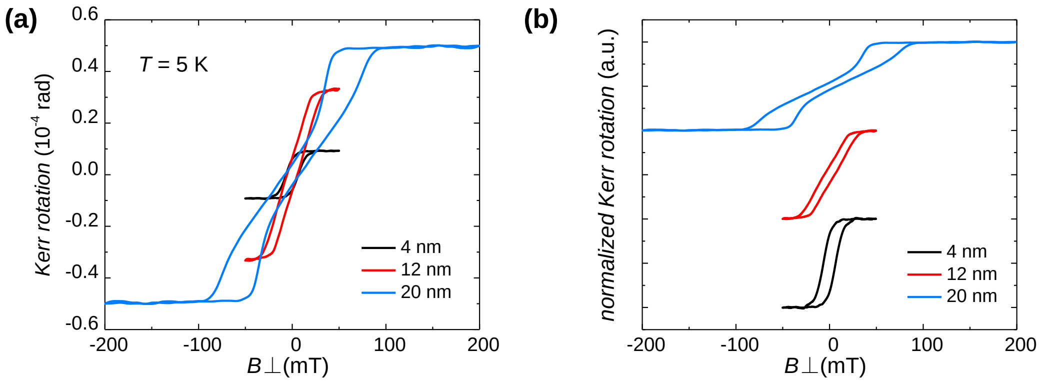

Supplementary Fig. 15. Magnetic characterizations of nanoflake $Cr_2Ge_2Te_6$ by MOKE. (a) Kerr angle measured at 5 K of three $Cr_2Ge_2Te_6$ nanoflakes with different thicknesses (black: 4 nm, red: 12 nm, blue: 20 nm). **(b)** Renormalized Kerr angle obtained from the data in **(a)** (stacked for clarity).

Supplementary Note 6. Barrier thickness dependence of Josephson junction parameters

The transport property of the tunnel junctions can be obtained from the thickness dependence of junction resistance area product R_nA, and the result of our experiments is shown in Supplementary Fig. 16a containing all the CGT devices from the Josephson coupling state to the tunneling region as defined in the supplementary information. Samples within the thinner barrier region but still with residual resistance are also included. The normal state resistances R_n of all the junctions were measured at 8 K above T_c. We find an exponential increase of R_nA fitted to an $\exp(d_F/t)$ with the barrier thickness d_F ranging from 6-15 nm (guided by the red dashed line), where the characteristic quasiparticle tunnel length t is calculated to be 2 nm, slightly larger than the value obtained by Idzuchi et al[27] of 1.3 nm. However, the normalized barrier resistance constant is around 3-4 $\Omega\ \mu m^2$, which is much lower than their result of 340 $\Omega\cdot\mu m^2$. The relatively small value of our junctions may partially be attributed to the decrease of the semiconducting gap of thicker CGT than the ultrathin flakes as 1-6L they used, yet, the leakage of charge carriers across the barrier is more likely to be the main cause. Furthermore, we summarize the thickness dependence of the R_nA product, the critical current density J_c and the I_cR_n product of the only JJs whose resistances have exactly dropped to zero at 2 K in our PPMS instruments. As in Supplementary Fig. 16b, except for the thickest barrier sample with an enhanced supercurrent mentioned before in the main text, we find that the J_c decreases with increasing d_F in

the rest devices, but the I_cR_n product does not show a monotonic dependence. Besides, neither the R_nA product nor the J_c shows an evidently exponential change as expected for an effective insulating barrier, leading to the moderate variations of the I_cR_n product. The relatively slow decrease of the J_c can also support the assumption of charge leakage to explain why our CGT barrier is not so insulating as it should be. Finally, we have to admit the individual differences in our samples reflected in their transport properties, as it is hard to control all the variables in the fabrication process of these vdW JJs, and we are also inclined to ascribe some observations above to an unphysical excuse. At the same time, the data based on these 5 JJs are not adequate to entirely reveal the superconducting nature beneath, therefore more detailed experiments are needed especially for the ultrathin CGT barriers in further studies.

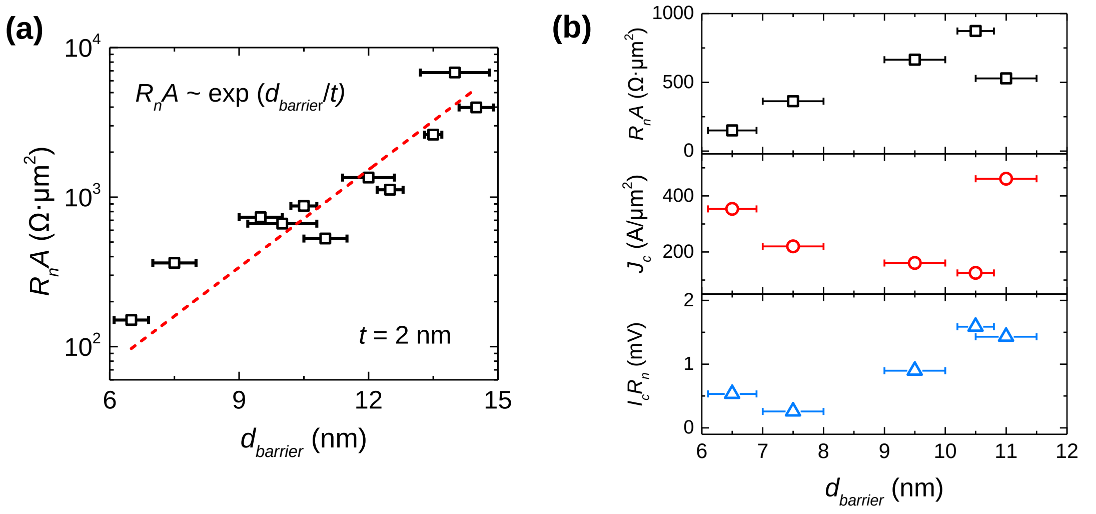

Supplementary Fig. 16. Barrier thickness dependence of the parameters. (a) The resistance area R_nA product of the junction (black rectangles) with different barrier thicknesses. The fitting result of the data by the exponential relation is indicated by the red dashed line. **(b)** The R_nA product (black rectangles), critical current density J_c (red circles), and characteristic voltage I_cR_n product (blue triangles) of the junction with different barrier thickness for absolute JJs whose resistances drop to exactly zero at 2 K.

Supplementary Note 7. Characterizations of single critical current due to the high damping environment in our Josephson junctions

The Josephson energy profile of a φ-JJ as a double-well potential should give the ground states phases of ±φ. Upon applying a bias current to the JJ corresponding to a tilt of the potential, the phase could be trapped either in the +φ or -φ state following a specific bias sweeping sequence[28,29]. When switching the JJ from the zero-voltage state to the voltage-state, the required tilt for the escape of the phase out of the potential well depends on the initial state (+φ or -φ) of the junction, at a higher I_{c+} or a lower I_{c-} alternatingly as we could get in positive bias currents. Goldobin et al[29–31] have demonstrated the possibility to use $I_{c\pm}$ for the readout of the ±φ state. However,

the I_{c-} can only be observed together with the I_{c+} for low damping[30,32] (underdamped JJ) allowing a stationary phase motion, because the probability to find the phase trapped in a certain state is nearly constant for ±φ. In contrast, for the regime of high damping (overdamped JJ), the retrapping process is deterministic to predict the particular destination well of the Josephson phase, therefore only the higher I_{c+} can be measured. Due to non-negligible electronic noises in the external circuits of our instruments, as we have suggested in the main text, we were not able to evaluate the damping in our JJs accurately[33,34], since their IVCs show tiny hystereses in the process of switching and retrapping like high damping JJs. For device #02 in which the unconventional Fraunhofer pattern was observed, we used two schemes to measure the two switching currents at zero magnetic field as predicted in a φ-JJ. The initial state of the JJ is prepared by sweeping from a large positive bias to zero. According to the comparison in Supplementary Fig. 17a, a switching current I_{c0} got by sweeping from zero to positive bias is almost identical to the other one I_{c-}, got by sweeping from large negative bias to positive bias. In other words, we failed to observe the evidence of a φ-JJ following this method because of the high damping circumstance.

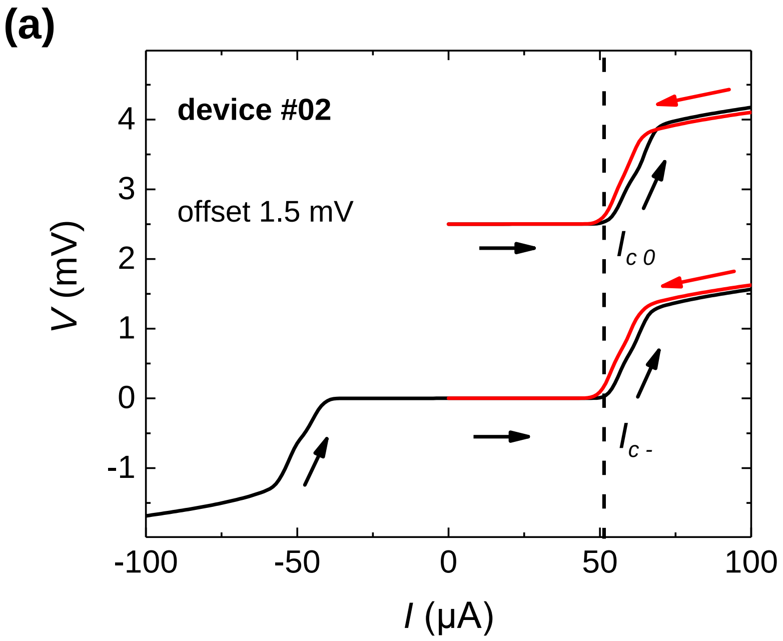

Supplementary Fig. 17. Sweeping schemes dependence of switching currents of device #02 at zero magnetic field. (a) A switching current I_{c0} is obtained by sweeping from large positive bias to zero bias and then back to positive bias (added 1.5 mV offset for clarity), while another switching current I_{c-} is obtained by sweeping from large negative bias to positive currents. The comparison of these two currents labeled by the black dashed line gives the almost identical value, representing the failure in determining the φ-JJ due to the high damping circumstance.

Supplementary Note 8. Derivations of the magnetic field dependences of the flux

and the interlayer's magnetization

Following the procedure called Josephson magnetometry[35,36], the experimental $I_c(B)$ or $R(B)$ curves can be transformed into the $M(B)$ curves, as the positions of the maximum (minimum) and minimum (maximum) of the I_c (R) are determined by the relations

$$I_c(B)\text{:}\ \ \Phi^{(min)} = m\Phi_0, \Phi^{(max)} = \left(n+\frac{1}{2}\right)\Phi_0, \tan[\frac{\pi\Phi^{(max)}}{\Phi_0}] = \frac{\pi\Phi^{(max)}}{\Phi_0}$$

$$R(B)\text{:}\ \ \Phi^{(min)} = \left(n+\frac{1}{2}\right)\Phi_0, \Phi^{(max)} = m\Phi_0, \tan[\frac{\pi\Phi^{(min)}}{\Phi_0}] = \frac{\pi\Phi^{(min)}}{\Phi_0}$$

where m and n are integers.

As the magnetic homogeneity of the interlayer is satisfied, the magnetic flux Φ through the junction is the sum of the flux Φ_M due to the ferromagnetic interlayer's magnetization M and the external field flux Φ_H

$$\Phi = \Phi_{\mathrm{M}} + \Phi_{\mathrm{H}} = 4\pi MWd_{\mathrm{F}} + \Phi_{\mathrm{H}}$$

where W is the junction width perpendicular to the magnetic field, d_F is the thickness of the ferromagnetic barrier and Φ_H is proportional to the magnetic field. When we get the $R(B)$ curves as shown in the upper panel in Fig. 3a, we first define the central lobe as the zero-order index (n = 0), and the successive peaks and valleys are determined as the positions of the index equal to ±1, ±3/2, ±2…… Using these relations we can transform $R(B)$ into $\Phi(B)$ dependence as the result presented in the lower panel of Fig. 3a.

The $\Phi(B)$ curves can further be transformed into $M(B)$ curves by subtracting the external field flux term and the results are compared in Supplementary Fig. 18a. The magnitudes of the interlayer's remanences and the saturation magnetizations can be read from the $M(B)$ curves. However due to the nonuniformity of periods of this device especially at high fields, rough approximations of them are expected rather than the exact estimations based on our data, therefore the direct derivations as the $\Phi(B)$ curves are preferred in the main text.

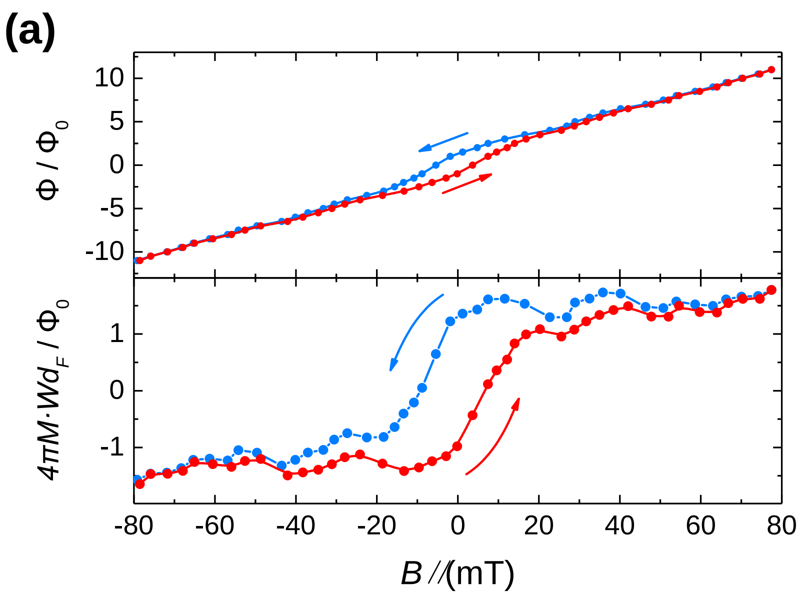

Supplementary Fig. 18. Extracted $\Phi(B)$ and $M(B)$ from $R(B)$ in device #01. (a)

Magnetic field dependence of trapped flux in the junction (upper), with each point representing integer or half-integer Φ_0 corresponding to the maximum or minimum in $R(B)$. The barrier's magnetization (lower) curves $M(B)$ are obtained from the data in $\Phi(B)$.

Supplementary References

1. Senapati, K., Blamire, M. G. & Barber, Z. H. Spin-filter Josephson junctions. *Nat. Mater.* **10**, 849–852 (2011).
2. Chang, J.-J. Fiske steps in Josephson junctions. *Phys. Rev. B* **28**, 1276–1280 (1983).
3. Pfeiffer, J. *et al.* Static and dynamic properties of 0, π, and 0 − π ferromagnetic Josephson tunnel junctions. *Phys. Rev. B* **77**, 214507 (2008).
4. Petković, I., Aprili, M., Barnes, S. E., Beuneu, F. & Maekawa, S. Direct dynamical coupling of spin modes and singlet Josephson supercurrent in ferromagnetic Josephson junctions. *Phys. Rev. B* **80**, 220502 (2009).
5. Wild, G., Probst, C., Marx, A. & Gross, R. Josephson coupling and Fiske dynamics in ferromagnetic tunnel junctions. *Eur. Phys. J. B* **78**, 509–523 (2010).
6. Tinkham, M. *Introduction to superconductivity*. (Courier Corporation, 2004).
7. Khestanova, E. *et al.* Unusual Suppression of the Superconducting Energy Gap and Critical Temperature in Atomically Thin $NbSe_2$. *Nano Lett.* **18**, 2623–2629 (2018).
8. Sohn, E. *et al.* An unusual continuous paramagnetic-limited superconducting phase transition in 2D $NbSe_2$. *Nat. Mater.* **17**, 504–508 (2018).
9. Chen, C. D., Yao, Y. D., Lee, S. F. & Shyu, J. H. Magnetoresistance study in Co–Al–Co and Al–Co–Al double tunneling junctions. *J. Appl. Phys.* **91**, 7469–7471 (2002).
10. Bose, S. K. & Budhani, R. C. Robust coupling of superconducting order parameter in a mesoscale NbN–Fe–NbN epitaxial structure. *Appl. Phys. Lett.* **95**, 042507 (2009).
11. Fang, Y., Wu, S., Zhu, Z.-Z. & Guo, G.-Y. Large magneto-optical effects and magnetic anisotropy energy in two-dimensional $Cr_2Ge_2Te_6$. *Phys. Rev. B* **98**, 125416 (2018).
12. Lohmann, M. *et al.* Probing Magnetism in Insulating $Cr_2Ge_2Te_6$ by Induced Anomalous Hall Effect in Pt. *Nano Lett.* **19**, 2397–2403 (2019).
13. Gong, C. *et al.* Discovery of intrinsic ferromagnetism in two-dimensional van der Waals crystals. *Nature* **546**, 265–269 (2017).
14. Kooy, C. Experimental and theoretical study of the domain configuration in thin layers of $BaFe_{12}O_{19}$. *Philips Res Repts* **15**, 7 (1960).
15. Kaplan, B. & Gehring, G. A. The domain structure in ultrathin magnetic films. *J. Magn. Magn. Mater.* **128**, 111–116 (1993).
16. Kim, K.-J. & Choe, S.-B. Analytic theory of wall configuration and depinning mechanism in magnetic nanostructure with perpendicular magnetic anisotropy. *J. Magn. Magn. Mater.* **321**, 2197–2199 (2009).
17. Zhang, X. *et al.* Magnetic anisotropy of the single-crystalline ferromagnetic insulator $Cr_2Ge_2Te_6$. *Jpn. J. Appl. Phys.* **55**, 033001 (2016).

18. Lin, Z. *et al.* Pressure-induced spin reorientation transition in layered ferromagnetic insulator $Cr_2Ge_2Te_6$. *Phys. Rev. Mater.* **2**, 051004 (2018).
19. Liu, W. *et al.* Anisotropic magnetoresistance behaviors in the layered ferromagnetic $Cr_2Ge_2Te_6$. *J. Phys. Appl. Phys.* **53**, 025101 (2020).
20. Mogi, M. *et al.* Ferromagnetic insulator $Cr_2Ge_2Te_6$ thin films with perpendicular remanence. *APL Mater.* **6**, 091104 (2018).
21. Selter, S., Bastien, G., Wolter, A. U. B., Aswartham, S. & Büchner, B. Magnetic anisotropy and low-field magnetic phase diagram of the quasi-two-dimensional ferromagnet $Cr_2Ge_2Te_6$. *Phys. Rev. B* **101**, 014440 (1–10) (2020).
22. Zhang, T. *et al.* Laser-induced magnetization dynamics in a van der Waals ferromagnetic $Cr_2Ge_2Te_6$ nanoflake. *Appl. Phys. Lett.* **116**, 223123 (2020).
23. Wang, Z. *et al.* Electric-field control of magnetism in a few-layered van der Waals ferromagnetic semiconductor. *Nat. Nanotechnol.* **13**, 554–559 (2018).
24. Han, M.-G. *et al.* Topological Magnetic-Spin Textures in Two-Dimensional van der Waals $Cr_2Ge_2Te_6$. *Nano Lett.* **19**, 7859–7865 (2019).
25. Guo, T. *et al.* Multiple domain structure and symmetry types in narrow temperature and magnetic field ranges in layered $Cr_2Ge_2Te_6$ crystal measured by magnetic force microscope. *Mater. Charact.* **173**, 110913 (2021).
26. Li, Y. F. *et al.* Electronic structure of ferromagnetic semiconductor $CrGeTe_3$ by angle-resolved photoemission spectroscopy. *Phys. Rev. B* **98**, 125127(1–6) (2018).
27. Idzuchi, H. *et al.* Unconventional supercurrent phase in Ising superconductor Josephson junction with atomically thin magnetic insulator. *Nat. Commun.* **12**, 5332 (2021).
28. Sickinger, H. *et al.* Experimental Evidence of a φ Josephson Junction. *Phys. Rev. Lett.* **109**, 107002 (2012).
29. Goldobin, E. *et al.* Memory cell based on a φ Josephson junction. *Appl. Phys. Lett.* **102**, 242602 (2013).
30. Goldobin, E., Koelle, D., Kleiner, R. & Buzdin, A. Josephson junctions with second harmonic in the current-phase relation: Properties of φ junctions. *Phys. Rev. B* **76**, 224523 (2007).
31. Goldobin, E., Koelle, D., Kleiner, R. & Mints, R. G. Josephson Junction with a Magnetic-Field Tunable Ground State. *Phys. Rev. Lett.* **107**, 227001 (2011).
32. Menditto, R. *et al.* Phase retrapping in a φ Josephson junction: Onset of the butterfly effect. *Phys. Rev. B* **93**, 174506 (2016).
33. Martinis, J. M. & Kautz, R. L. Classical phase diffusion in small hysteretic Josephson junctions. *Phys. Rev. Lett.* **63**, 1507–1510 (1989).
34. Kautz, R. L. & Martinis, J. M. Noise-affected I-V curves in small hysteretic Josephson junctions. **42**, 9903–9938 (1990).
35. Bol'ginov, V. V., Stolyarov, V. S., Sobanin, D. S., Karpovich, A. L. & Ryazanov, V. V. Magnetic switches based on Nb-PdFe-Nb Josephson junctions with a magnetically soft ferromagnetic interlayer. *JETP Lett.* **95**, 366–371 (2012).
36. Iovan, A., Golod, T. & Krasnov, V. M. Controllable generation of a spin-triplet supercurrent in a Josephson spin valve. *Phys. Rev. B* **90**, 134514 (2014).
